# Crystal structure of histone deacetylase 6 complexed with (*R*)-lipoic acid, an essential cofactor in central carbon metabolism

**DOI:** 10.1016/j.jbc.2023.105228

**Published:** 2023-09-12

**Authors:** Paris R. Watson, Juana Goulart Stollmaier, David W. Christianson

**Affiliations:** Roy and Diana Vagelos Laboratories, Department of Chemistry, University of Pennsylvania, Philadelphia, Pennsylvania, United States

**Keywords:** HDAC, metalloenzyme, enzyme inhibitor, protein crystallography, isothermal titration calorimetry

## Abstract

The enzyme cofactor (*R*)-lipoic acid plays a critical role in central carbon metabolism due to its catalytic function in the generation of acetyl-CoA, which links glycolysis with the tricarboxylic acid cycle. This cofactor is also essential for the generation of succinyl CoA within the tricarboxylic acid cycle. However, the biological functions of (*R*)-lipoic acid extend beyond metabolism owing to its facile redox chemistry. Most recently, the reduced form of (*R*)-lipoic acid, (*R*)-dihydrolipoic acid, has been shown to inhibit histone deacetylases (HDACs) with selectivity for the inhibition of HDAC6. Here, we report the 2.4 Å-resolution X-ray crystal structure of the complex between (*R*)-dihydrolipoic acid and HDAC6 catalytic domain 2 from *Danio rerio*, and we report a dissociation constant (K_D_) of 350 nM for this complex as determined by isothermal titration calorimetry. The crystal structure illuminates key affinity determinants in the enzyme active site, including thiolate-Zn^2+^ coordination and S-π interactions in the F583-F643 aromatic crevice. This study provides the first visualization of the connection between HDAC function and the biological response to oxidative stress: the dithiol moiety of (*R*)-dihydrolipoic acid can serve as a redox-regulated pharmacophore capable of simultaneously targeting the catalytic Zn^2+^ ion and the aromatic crevice in the active site of HDAC6.

The well-known enzyme cofactor (*R*)-lipoic acid ((6*R*)-6,8-dithiooctanoic acid) plays a critical role in central carbon metabolism due to the facile redox chemistry of its disulfide moiety (Fig. 1) (1, 2, 3). A prime example includes catalysis by the pyruvate dehydrogenase complex that links glycolysis with the tricarboxylic acid (TCA) cycle (4, 5, 6, 7). (*R*)-Lipoic acid is covalently tethered to a flexible lysine residue in the E2 subunit of pyruvate dehydrogenase and in this context is referred to as (*R*)-lipoamide. When bound to the E1 subunit, (*R*)-lipoamide is acetylated to yield the reduced form of the cofactor, acetyl-(*R*)-dihydrolipoamide, which then swings back to the E2 subunit where it acetylates coenzyme A (CoA) to yield acetyl-CoA and free (*R*)-dihydrolipoamide. Acetyl-CoA then enters the TCA cycle, and (*R*)-dihydrolipoamide is oxidized in the E3 subunit to regenerate (*R*)-lipoamide. The same reaction sequence is catalyzed by the α-ketoglutarate dehydrogenase complex in the TCA cycle, where E2-linked (*R*)-lipoamide is required for the generation of succinyl-CoA (8, 9).

**Figure 1:**
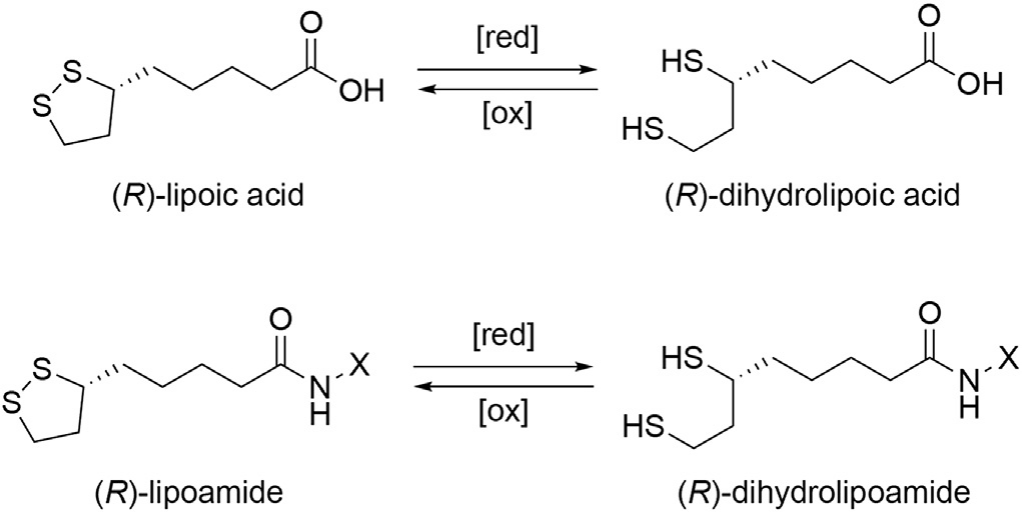
**Molecular structure of (*R*)-lipoic acid.** The oxidized and reduced states of (*R*)-lipoic acid are shown (top); [*red*] = reduction, [*ox*] = oxidation. When attached to the side chain of a lysine residue (X) in the E2 subunit of pyruvate dehydrogenase, the cofactor is referred to as (*R*)-lipoamide in the oxidized state and (*R*)-dihydrolipoamide in the reduced state (*bottom*); the cofactor is also referred to as (*R*)-lipoamide when amidated to form a simple carboxamide (X = H). The C8 thiol group of (*R*)-dihydrolipoamide undergoes reversible acetylation in the biosynthesis of acetyl-CoA. Oxidation of (*R*)-dihydrolipoamide in the E3 subunit of pyruvate dehydrogenase regenerates the disulfide linkage of (*R*)-lipoamide for another catalytic turnover.

Beyond central carbon metabolism, free (*R*)-lipoic acid (or the racemic mixture of free (*R*)- and (*S*)-lipoic acid, designated "(*R/S*)") is involved in diverse biological processes. It is an antioxidant that can be used to manage oxidative stress in chronic human disease, such as diabetic neuropathy (10). When used in this manner, (*R*)-lipoic acid has been shown to lower blood triglyceride concentrations, although this effect has not been universally observed (11, 12, 13). Due to its neuroprotective antioxidant effects, (*R*)-lipoic acid is considered as a possible nutraceutical for Alzheimer's disease therapy (14); moreover, (*R*)-lipoic acid is efficacious in animal models of neurodegeneration as well as other oxidative stress-related disorders such as ischemia-reperfusion injury and cataract formation (15, 16).

In a 2023 *Nature Communications* paper, Lechner *et al.* (17) report that (*R*)-dihydrolipoic acid is an inhibitor of the Zn^2+^-dependent histone deacetylases (HDACs), with 5 to 15-fold preferential selectivity for inhibition of HDAC6 (EC_50_ = 1 μM) over other isozymes. Neither the oxidized form of the inhibitor, (*R*)-lipoic acid, nor its stereoisomer (*S*)-dihydrolipoic acid, are effective inhibitors. HDAC6 is a class IIb enzyme (18) predominantly localized in the cell cytosol (19, 20, 21, 22), where it serves as a tubulin deacetylase and Tau deacetylase (23, 24). The cell cytosol is a reducing environment, so the disulfide linkage of (*R*)-lipoic acid will exist here predominantly in the reduced form as (*R*)-dihydrolipoic acid with C6 and C8 thiol groups (25). Accordingly, Lechner *et al.* (17) hypothesize that one or both of the thiol groups coordinate to the catalytic Zn^2+^ ion in the active site of HDAC6 (as well as other HDAC isozymes), but they do not report a crystal structure to verify their hypothesis. Of note, contrasting proposals suggest that the carboxylate group of (*R*)-lipoic acid serves as the Zn^2+^-binding moiety (26, 27). Ambiguities regarding the inhibitory binding mode of (*R*)-dihydrolipoic acid must be resolved to fully understand the biological activity of this critical enzyme cofactor.

Here, we resolve these ambiguities by reporting the 2.4 Å resolution X-ray crystal structure of the complex between (*R*)-dihydrolipoic acid and HDAC6 catalytic domain 2 from *Danio rerio* (zebrafish). We also report the dissociation constants (K_D_) of (*R/S*)-dihydrolipoic acid, (*R*)-lipoic acid, (*R/S*)-dihydrolipoamide (X = H in Fig. 1), and (*S*)-dihydrolipoic acid as measured using isothermal titration calorimetry (ITC). The naturally occurring (*R*)-dihydrolipoic acid stereoisomer exhibits the tightest binding with K_D_ = 350 nM, and the crystal structure of the HDAC6 complex reveals that critical enzyme-inhibitor interactions include C8–S^–^•••Zn^2+^ coordination and C6-SH•••aromatic interactions in the active site.

## Results

### Crystal structure

**Figure 2:**
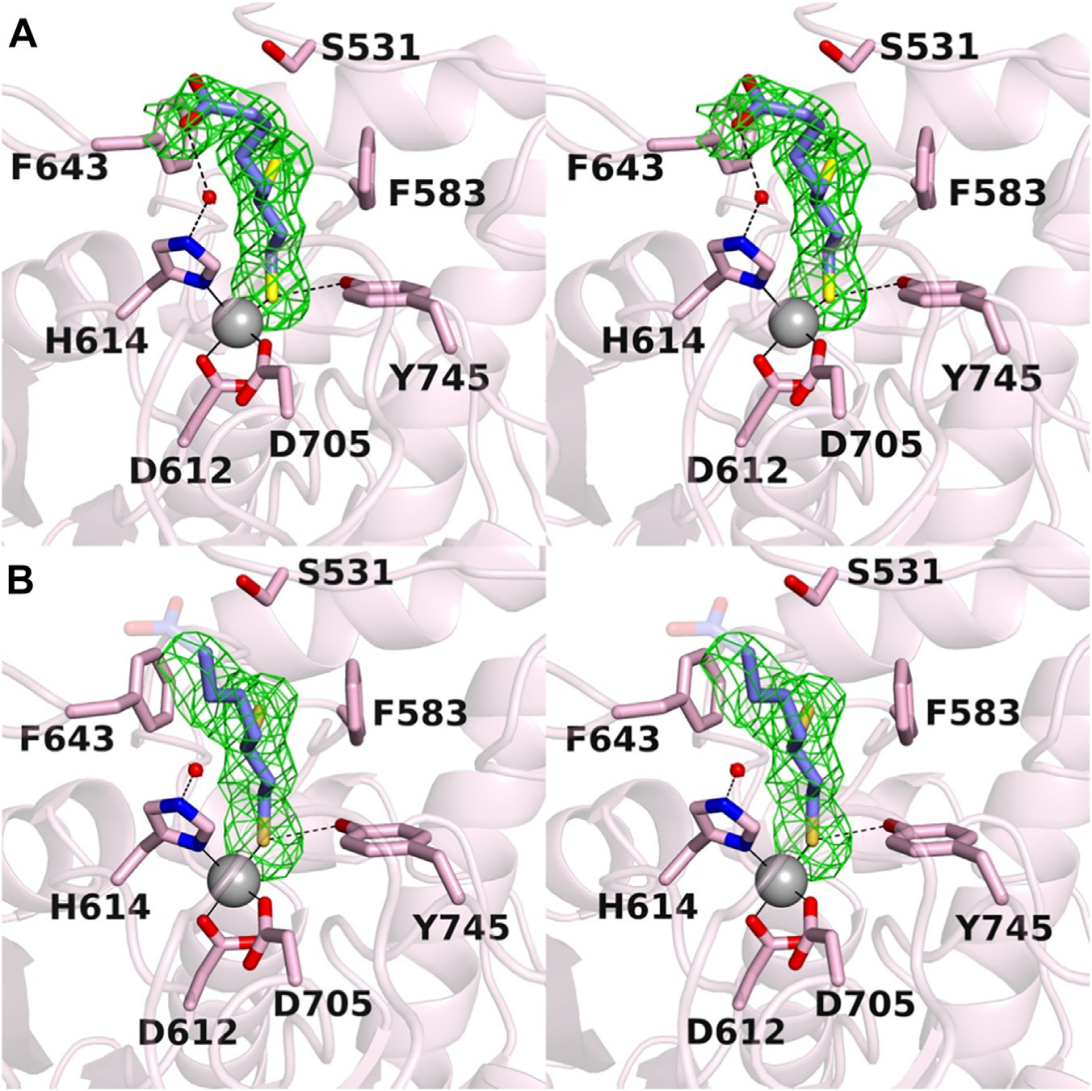
**Stereo views of electron density maps showing (*R*)-dihydrolipoic acid bound in the HDAC6 active site.** Atomic color codes are as follows: C = *mauve* (HDAC6) or *blue* ((*R*)-dihydrolipoic acid), N = *blue*, O = *red*, Zn^2+^ = *gray sphere*; metal coordination and hydrogen bond interactions are indicated by *solid* and *dashed black lines*, respectively. Selected active site residues are shown as *stick figures* and labeled; of note, these residues are strictly conserved between zebrafish and human HDAC6. *A*, polder omit map contoured at 3.2σ showing (*R*)-dihydrolipoic acid bound in the active site of monomer A. *B*, polder omit map contoured at 3.2σ showing (*R*)-dihydrolipoic acid bound in the active site of monomer B. Here, the terminal carboxylate group is disordered and shown as a "ghost" image for reference; these atoms are not included in the final model. HDAC6, histone deacetylase 6.

The structure of the HDAC6–(*R*)-lipoic acid complex was determined at 2.4 Å resolution in space group *P*2_1_2_1_2_1_ with two independent monomers A and B in the asymmetric unit. The binding of (*R*)-dihydrolipoic acid to HDAC6 does not trigger any significant structural changes in either monomer, and the root-mean-square deviation of 325 Cα atoms is 0.189 between monomer A in the inhibitor-bound and unliganded (PDB 5EEM) (21) enzyme structures. Electron density maps show that the inhibitor binds in the active site with only the C8 thiol group coordinated to the catalytic Zn^2+^ ion (average S•••Zn^2+^ distance = 2.35 Å) (Fig. 2). Coordination of a thiol group to Zn^2+^ lowers the pKa of the thiol group from ∼8.5 to ∼6 and thereby facilitates ionization (28), yielding a potent thiolate-Zn^2+^ charge–charge interaction. The Zn^2+^-bound thiolate is also stabilized by a hydrogen bond with catalytic tyrosine Y745.

The C6 thiol group of (*R*)-dihydrolipoic acid resides in an aromatic cleft formed by F583 and F643, where it engages in S-π interactions (29, 30). The C6 sulfur atom is located 3.7 Å and 4.0 Å from the aromatic ring centroids of F583 and F643, respectively, in monomer A. This is the first example of an S-π interaction in the aromatic crevice of HDAC6, where more usually the aromatic linker groups of inhibitors typically pack (21, 31, 32, 33). Finally, in view of the typical carboxylic acid pKa of ∼4, the carboxylic acid moiety of (*R*)-dihydrolipoic acid would be ionized as the negatively charged carboxylate at the pH of the crystal structure determination (pH 7.5). This carboxylate group extends out of the active site: in monomer A, the carboxylate group forms a hydrogen bond with a water molecule that, in turn, forms a hydrogen bond with Zn^2+^ ligand H614 (Fig. 2*A*). In monomer B, the carboxylate group is disordered (Fig. 2*B*). Clearly, the carboxylate group does not engage in direct Zn^2+^ coordination in either monomer, though the carboxylate-H_2_O-H614 hydrogen bond network in monomer A might be viewed as an indirect interaction with Zn^2+^.

It should be noted that Lechner *et al.* (17) utilized full-length human HDAC6 or catalytic domain 2 from human HDAC6 for inhibitor assay experiments, whereas zebrafish HDAC6 catalytic domain 2 was utilized for our X-ray crystallographic and ITC studies. We previously determined the structure of human HDAC6 catalytic domain 2 and showed that its active site structure is essentially identical to that of zebrafish HDAC6 catalytic domain 2 (21). Specifically, all active site residues shown in Figure 2 are strictly conserved between the zebrafish and human enzymes. Since zebrafish HDAC6 catalytic domain 2 is more readily crystallized, and crystals usually diffract to higher resolution compared with the human enzyme, the zebrafish enzyme serves as a valid surrogate for understanding structure–function relationships for the human enzyme.

### Isothermal titration calorimetry

**Figure 3:**
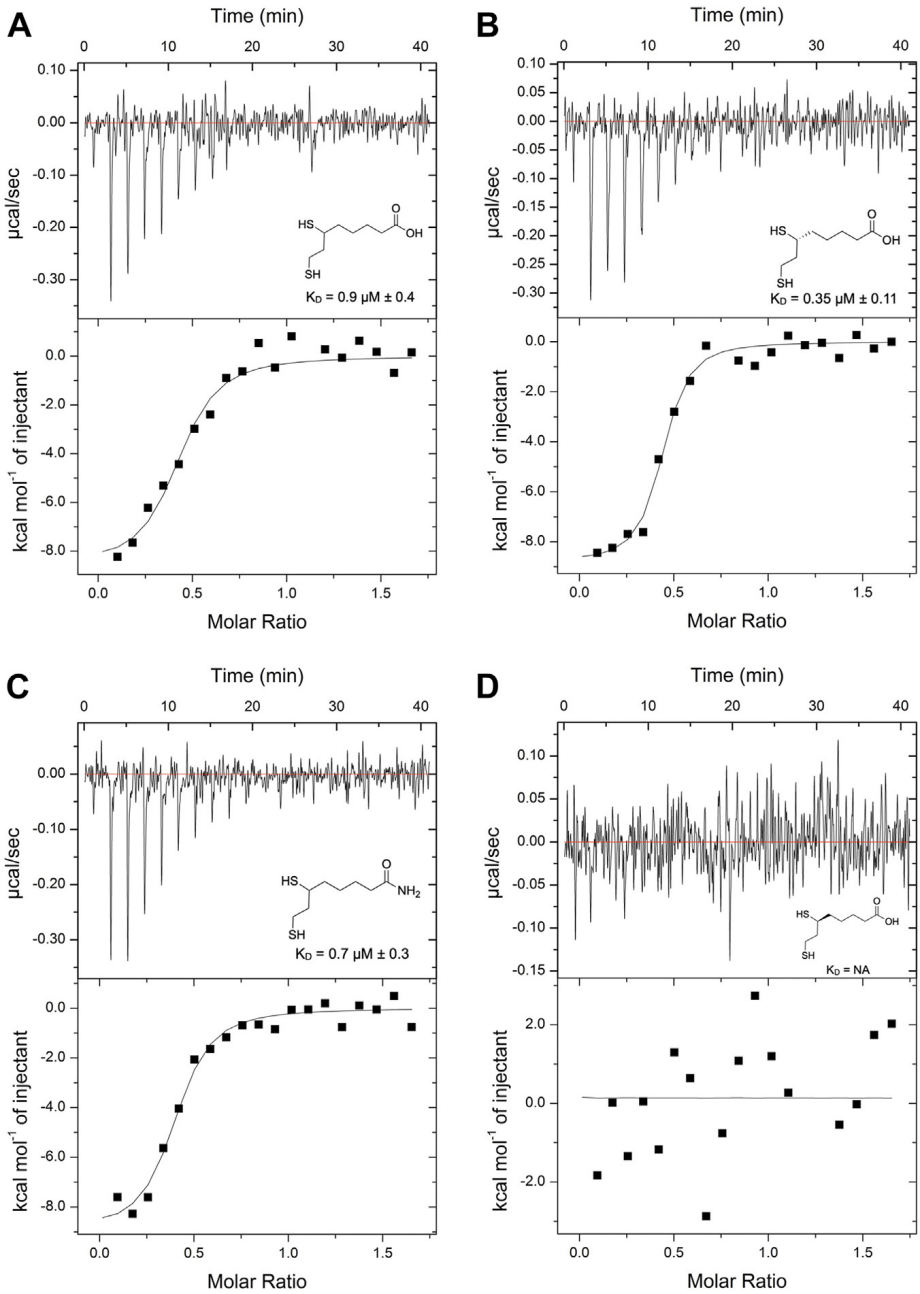
**Isothermal titration calorimetry.***A*–*D*, enthalpograms for the binding of (*A*) (*R/S*)-dihydrolipoic acid, (*B*) (*R*)-dihydrolipoic acid, (*C*) (*R/S*)-dihydrolipoamide, and (*D*) (*S*)-dihydrolipoic acid to HDAC6. HDAC6, histone deacetylase 6.

To gain further insight into the binding affinity of lipoic acid derivatives to HDAC6, we studied the thermodynamics of enzyme−inhibitor association using ITC. The dissociation constants (K_D_) determined for each enzyme–inhibitor pair (Fig. 3) follow a similar trend to the reported EC_50_ values (17). The preferred stereoisomer, (*R*)-lipoic acid, binds with a K_D_ value approximately one-half that of the K_D_ values measured for racemic (*R/S*)-lipoic acid and (*R/S*)-lipoamide. This is consistent with the binding of only the (*R*)-stereoisomer from the racemic mixture; binding was not detected for the (*S*)-stereoisomer.

## Discussion

Perhaps the most prominent feature in the crystal structure of the HDAC6–(*R*)-lipoic acid complex is the coordination of the inhibitor C8 thiolate group to the catalytic Zn^2+^ ion. A handful of crystal structures have been determined of deacetylases complexed with inhibitors bearing thiol Zn^2+^-binding groups, such as the HDAC8–Largazole complex (34, 35) and HDAC6 complexes with thiol-containing macrocyclic octapeptides (36, 37). Upon coordination to Zn^2+^, the pKa of the thiol group is lowered so as to facilitate ionization, thereby yielding a potent thiolate-Zn^2+^ charge–charge interaction. The ideal geometric parameters for thiolate-Zn^2+^ coordination were first outlined by Chakrabarti through analysis of cysteine-metal ion coordination interactions in the Protein Data Bank (38): the average S^–^•••Zn^2+^ separation is 2.1 Å, the average C–S^–^•••Zn^2+^ angle is 108°, and the preferred C–C–S^–^•••Zn^2+^ dihedral angles are 180° and ± 90°. In monomers A and B of the HDAC6–(*R*)-lipoic acid complex, the S^–^•••Zn^2+^ separations are 2.3 Å and 2.4 Å, the C–S^–^•••Zn^2+^ angles are 117° and 122°, and the C–C–S^–^•••Zn^2+^ dihedral angles are 56° and 37°, respectively. While these parameters deviate from the ideal parameters outlined above, they nonetheless allow for high affinity binding.

**Figure 4:**
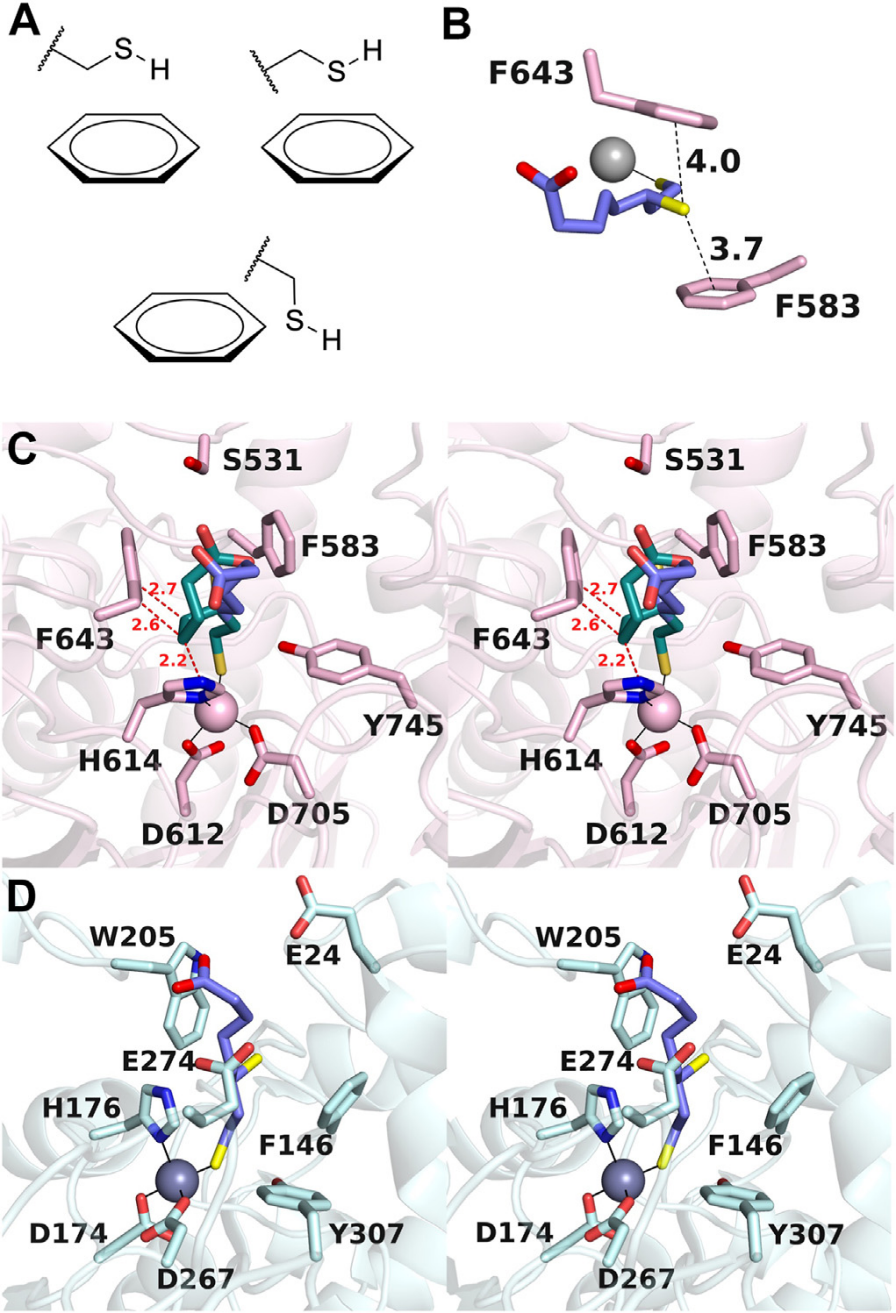
**S-π interactions.***A*, three possible orientations for S-H---π interactions. *B*, distance of S-π interactions between HDAC6 and the C-6 thiol group of R-α-dihydrolipoic acid in monomer A. The C-6 thiol group is located nearly directly over the centroids of each aromatic ring. *C*, stereo view of a model of (*S*)-dihydrolipoamide (C = *turquoise*) binding in the HDAC6 active site prepared from the structure of the (*R*)-dihydrolipoamide (C = *blue*) binding mode in monomer A. Steric clashes with (*S*)-dihydrolipoamide are indicated by *red dotted lines* and distances. *D*, stereo view of a model of (*R*)-dihydrolipoamide bound in the active site of HDAC10, in which the binding conformation of the inhibitor in HDAC6 monomer A was mapped onto the coordinates of HDAC10. HDAC6, histone deacetylase 6; HDAC10, histone deacetylase 10.

A second prominent structural feature of the HDAC6–(*R*)-dihydrolipoic acid complex involves the C6 thiol group, which engages in S-π interactions in the F583-F643 aromatic crevice. There are three possible orientations for energetically favorable S-π interactions (Fig. 4*A*) (39), two of which are thiol interactions with the face of the aromatic ring. The C6 thiol group of (*R*)-dihydrolipoic acid clearly interacts with the faces of the F583 and F643 aromatic rings (Fig. 4*B*). The geometries of thiol-aromatic interactions with F583 and F643 are generally consistent with energetically favorable interactions observed between the thiol side chain of cysteine and aromatic residues in an analysis of protein structures contained in the PDB, as well as molecular orbital calculations showing that the positioning of an alkylthiol moiety above an aromatic ring centroid is most preferable (30, 40).

Notably, molecular recognition of the thiol groups dictates stereoselectivity for the binding of (*R*)-dihydrolipoic acid over the binding of (*S*)-dihydrolipoic acid. If the (*S*)-stereoisomer were to bind with the C8 thiolate coordinated to Zn^2+^ and the C6 thiol bound in the aromatic crevice, our modeling studies suggest that the C5 and C4 methylene groups of (*S*)-dihydrolipoic acid would sterically clash with H614 and F643 (Fig. 4*C*).

Interestingly, Lechner *et al.* (17) show that (*R/S*)-dihydrolipoamide (here, with the carboxylate group amidated to form a neutral carboxamide, such that R = H in Fig. 1), but not (*R/S*)-dihydrolipoic acid, is an inhibitor of the class IIb isozyme HDAC10. Like HDAC6, HDAC10 is enriched in the cytosol (41), but unlike HDAC6, HDAC10 is a polyamine deacetylase that exhibits narrow substrate specificity for the hydrolysis of *N*^8^-acetylspermidine (42). A negatively charged glutamate residue unique to the HDAC10 active site, E274, confers polyamine substrate specificity by engaging the positively charged secondary ammonium group of *N*^8^-acetylspermidine with water-mediated hydrogen bonds (43). If the HDAC6 binding conformation of (*R*)-dihydrolipoic acid is modeled into the active site of HDAC10, the carboxylate group of the inhibitor would reside close to the carboxylate group of E274 and also nearby E24, both of which are clearly repulsive electrostatic interactions (Fig. 4*D*). This explains why neutral (*R*)-dihydrolipoamide is a much better inhibitor of HDAC10, thereby verifying the proposal advanced by Lechner *et al.* (17) for HDAC10 inhibitory potency.

Notably, (*R*)-lipoamide is reported to be a substrate for HDAC11, which catalyzes the hydrolysis of the amide linkage with the tethered lysine side chain (44). HDAC11 is a lysine fatty-acid deacylase and can process a variety of fatty acid and cofactor conjugates with lysine residues (44, 45, 46). Thus, different HDAC isozymes may be involved in lipoic acid function in the cell.

With regard to the biological implications of (*R*)-lipoic acid inhibition of HDAC isozymes, Lechner *et al.* (17) report that both (*R*)-lipoic acid and (*R*)-lipoamide inhibit stress granule formation in cancer cells, an effect that has also been observed for other HDAC inhibitors such as Trichostatin and Tubastatin (47). Moreover, increased HDAC activity is observed following induction of oxidative stress (48), and HDAC6 is known to play a critical role in stress response (49). Accordingly, we conclude that the structure of the HDAC6–(*R*)-dihydrolipoic acid complex reported herein provides the first visualization of how (*R*)-lipoic acid might regulate HDAC function in the stress response.

## Experimental procedures

### Enzyme preparation

HDAC6 catalytic domain 2 from *D. rerio* (henceforth simply "HDAC6") was expressed and purified as previously detailed (50), with the modification of 0 mM imidazole in Buffer A.

### Isothermal titration calorimetry

Enthalpograms were measured using a MicroCal iTC 200 isothermal titration calorimeter (Malvern Panalytical). For (*R/S*)-lipoic acid (TCI Chemicals), (*S*)-lipoic acid (Toronto Research Chemicals), and (*R*)-lipoic acid (TCI Chemicals), 240 μM inhibitor was incubated for 3 h in size-exclusion (SE) buffer [50 mM Hepes (pH = 7.5), 100 mM KCl, 1 mM tris(2-carboxyethyl)phosphine (TCEP), 5% glycerol] and titrated against 30 μM HDAC6 in SE buffer. The (*R/S*)-lipoamide sample (Tochris Bioscience) was solubilized in DMSO and incubated for 3 h in SE buffer; 240 μM inhibitor was titrated against 30 μM HDAC6 in SE buffer with 5% DMSO. Twenty 2 μl-injections were made over 40 min, with constant stirring in the sample cell (750 r.p.m.) at 25 °C. Background was taken under same conditions without HDAC6 (inhibitor to buffer). Integration, reference subtraction, curve fitting, and figure generation were performed using Origin (OriginLab).

### Crystallography

The HDAC6 complex with (*R*)-lipoic acid was crystallized by the sitting-drop vapor diffusion method. Prior to inhibitor incubation, 4 μl of 40 mM (*R*)-lipoic acid was incubated with 0.4 μl of 1.0 M TCEP in 12.7 μl of buffer [50 mM Hepes (pH 7.5), 100 mM KCl, 5% glycerol (v/v), 1 mM TCEP] for 4 h. Following this, 22.9 μl of 17.5 mg/ml HDAC6 was added for a total volume of 40 μl. Final concentrations of key components were 10 mg/ml HDAC6, 4 mM (*R*)-lipoic acid, and 10 mM TCEP. A 200 nl drop of precipitant solution [0.2 M potassium citrate tribasic and 20% (w/v) polyethylene glycol 3350] was combined with a 200 nl drop of protein solution and equilibrated against 80 μl of precipitant solution in the well reservoir at 4 °C. Plate-like crystals formed in 2 days. Prior to data collection, crystals were flash-cooled in mother liquor supplemented with 20% ethylene glycol.

X-ray diffraction data were collected on the NSLS-II FMX beamline at Brookhaven National Laboratory. Data were indexed using XDS and scaled using Aimless (51, 52). The initial electron density map was phased by molecular replacement using Phaser (53) with the atomic coordinates of unliganded HDAC6 (PDB 5EEM) (21) as a search probe for rotation and translation function calculations (PDB 5EEM). The protein model was adjusted using Coot and refined in Phenix (54, 55). (*R*)-lipoic acid was fit to the electron density map in the later stage of refinement. Validation of the final model was performed with MolProbity (56). Data collection and refinement statistics are recorded in Table 1.

**Table 1:** Data collection and refinement statistics for the HDAC6–(*R*)-dihydrolipoic acid complex

Space group	*P*2_1_2_1_2_1_
a,b,c (Å)	74.89, 92.16, 96.67
α, β, γ (°)	90.0, 90.0, 90.0
R_merge_^a^	0.218 (0.655)
R_pim_^b^	0.106 (0.312)
CC_1/2_^c^	0.992 (0.965)
Redundancy	9.2 (9.9)
Completeness (%)	99.6 (100)
I/σ	8.7 (4.9)
Refinement	
Resolution (Å)	29.6–2.4 (2.49–2.40)
No. unique reflections	26,723 (2782)
R_work_/R_free_^d^	0.207/0.248 (0.232/0.307)
Number of atoms^e^	
Protein	5408
Ligand	27
Solvent	132
Average B factors (Å^2^)	
Protein	18
Ligand	21
Solvent	18
RMS deviations	
Bond lengths (Å)	0.003
Bond angles (°)	0.56
Ramachandran plot^f^	
Favored	96.71
Allowed	3.29
Outliers	0.00

Values in parentheses refer to the highest-resolution shell of data.

a R_merge_ = ∑_*h*_∑_*i*_*|*I_*i*_,_*h*_ − ⟨I⟩_*h*_|/∑_*h*_∑_*i*_I_*i*,*h*_, where ⟨I⟩_*h*_ is the average intensity calculated for reflection *h* from *i* replicate measurements.

b R_p.i.m._ = (∑_*h*_(1/(N-1))^1/2^∑_*i*_|I_*i*,*h*_ − ⟨I⟩_*h*_|)/∑_*h*_∑_*i*_I_*i*__,__*h*_, where N is the number of reflections, and ⟨I⟩_*h*_ is the average intensity calculated for reflection *h* from replicate measurements.

c Pearson correlation coefficient between random half-datasets.

d R_work_ = ∑||F_o_| − |F_c_||/∑|F_o_| for reflections contained in the working set. |F_o_| and |F_c_| are the observed and calculated structure factor amplitudes, respectively. R_free_ is calculated using the same expression for reflections contained in the test set held aside during refinement.

e Per asymmetric unit.

f Calculated with MolProbity.

## Data availability

Atomic coordinates and structure factor amplitudes of the HDAC6–(*R*)-lipoic acid complex have been deposited in the Protein Data Bank (www.rcsb.org) with accession code 8TQ0.

## Conflict of interest

The authors declare that they have no conflicts of interest with the contents of this article.
